# Research on the application of post competency training for new nurses: current status and considerations

**DOI:** 10.3389/fmed.2025.1537613

**Published:** 2025-04-28

**Authors:** Li Yingxia, Li Meiyao, Bao Jing, Zhao Aijuan

**Affiliations:** Department of Nursing, The First Affiliated Hospital of Jiamusi University, Jiamusi, China

**Keywords:** post competency, new recruit, training method, online training, nurse

## Abstract

The job competence of newly employed nurses is of crucial importance for nursing quality and patient safety. Nurse job competence emphasizes the comprehensive qualities of nursing knowledge, skills, attitudes, traits, and abilities that can achieve excellent work results in the actual nursing position and be competent for clinical nursing work. This article comprehensively analyzes relevant literature and elaborates on diverse training modalities for enhancing the job competency of newly recruited nurses. These encompass enhancements to conventional training approaches, simulation-based training, information-driven training, teamwork training, and scenario simulation cases. Additionally, it delves into the advantages, key implementation aspects, and associated challenges of these methods, with the objective of furnishing a reference for optimizing the training regimens of newly employed nurses.

## 1 Introduction

In the ever-evolving and highly dynamic realm of healthcare, the role played by nurses is of paramount importance in guaranteeing the provision of high-quality patient care. As stipulated in the Training Outline for New Nurses in China (Trial), new nurses refer to those who commence nursing positions subsequent to their graduation from colleges and universities and are required to undergo a 24-month training period (1). They are confronted with the arduous task of promptly acclimating to their professional roles and cultivating the requisite competencies. Nurse job competency encompasses a broad spectrum of knowledge, skills, attitudes, and capabilities that are indispensable for the effective execution of clinical duties (2, 3). The American Association of Colleges of Nursing (AACN) (4) has characterized job competency as a collection of multi-faceted and dynamic proficiencies that are molded through a diverse array of experiences and learning opportunities. In the context of Chinese nursing practice (5), it is accentuated as the proficiency to proficiently conduct clinical nursing tasks and attain outstanding performance outcomes. Given the escalating intricacy of healthcare systems and the burgeoning demands of patients, it is of utmost significance to investigate and implement efficacious training methodologies to augment the job competency of new nurses. The present review endeavors to conduct a comprehensive analysis of the existing state of post-competency training for new nurses and thereby establish a groundwork for prospective enhancements.

## 2 Training methods for new nurses

### 2.1 Traditional training methods and their enhancements

Traditional classroom lectures have long constituted a fundamental component in nurse training. Nevertheless, they frequently fall short in generating the requisite level of engagement for the effective transmission of complex information. To surmount this obstacle, the incorporation of multimedia elements, such as video demonstrations of procedures like cardiopulmonary resuscitation, can markedly augment the learning experience. When aptly employed, case analyses can furnish real-world contexts, rendering theoretical knowledge more accessible and comprehensible. For example, a research study conducted by Jangland et al. demonstrated that case-based learning enhanced the understanding and practical application of knowledge among novice nurses (6). Interactive elements such as group discussions and question-and-answer periods not only animate the classroom atmosphere but also stimulate critical thinking and promote active engagement.

Clinical rotation represents another crucial facet of traditional training. It acquaints new nurses with a diverse range of patient conditions and clinical scenarios, thereby broadening their comprehension of various medical and nursing practices. During rotations in departments such as the emergency room and intensive care unit, new nurses acquire the ability to adapt to distinct work environments and cultivate problem-solving capabilities. A meticulously designed rotation program, buttressed by research findings (7), can enhance the proficiency of new nurses in managing complex patient situations. However, the traditional one-to-one teaching model employed in clinical rotations may face challenges due to the variation in teaching quality among instructors. By standardizing the teaching process and implementing regular training and evaluation for teaching staff, this issue can be effectively addressed. Research by Chung, JYS. et al. argues that the utilization of a blended learning approach, which combines face-to-face training and online handover practice modules, has improved the communication skills and self-efficacy of nurses (8).

### 2.2 Manikin of mannequin in nurse training

The utilization of manikin dummies in nurse training has witnessed a growing prevalence. High-fidelity manikins are capable of replicating an extensive array of clinical scenarios, spanning from fundamental to intricate, thereby enabling new nurses to hone their skills within a risk-free milieu. For example, in a simulated cardiac arrest scenario, new nurses can execute chest compressions, operate defibrillators, administer medications, and concurrently practice communication with the patient’s family. Research has indicated that simulation training employing manikins can enhance the confidence and proficiency of new nurses in managing emergency situations (9). When combined with the standardized training of medical simulation teaching, it can augment the clinical job competence and teaching satisfaction of new nurses, yielding a remarkable teaching effect. In this manner, the emergency response capabilities of new nurses in critical situations are comprehensively enhanced, and they can acquire practical experience prior to attending to real patients.

### 2.3 Standardized patients in training

Standardized patients, who are trained individuals with the ability to precisely mimic patient symptoms and behaviors, occupy a vital position in the training of new nurses, particularly in relation to the development of communication and physical examination skills. Via interactions with standardized patients, new nurses are afforded the opportunity to practice obtaining medical histories, delivering patient education, and conducting physical examinations within a realistic context. This approach has been demonstrated to augment the communication and clinical skills of new nurses (10). The employment of standardized patients in conjunction with objective structured clinical examinations (OSCEs) has likewise proven efficacious in the evaluation and enhancement of the core competencies of new nurses.

### 2.4 Information-based training approaches

In the wake of the swift advancement of technology, online course platforms have emerged as highly valuable assets for nurse training. A multitude of hospitals and institutions presently present a diverse range of nursing courses in an online format, affording new nurses the flexibility to learn at their own pace and convenience. These courses encompass theoretical knowledge, practical skill demonstrations, as well as case analyses. For instance, an online course devised by Michel et al. (11) furnished comprehensive training in geriatric nursing and incorporated elements such as online testing and personalized feedback. The Small Private Online Course (SPOC) model has also attained significant popularity, empowering students to tailor their learning experiences and access top-notch educational resources. In a study by Tao et al. (12), Wang et al. (13), and Zhang et al. (14), it was ascertained that the SPOC model could enhance the learning outcomes and self-directed learning capabilities of nursing students. In the research carried out in China, the teaching model of SPOC maximizes the sharing of high-quality teaching resources, economizes on teaching costs, and alleviates the substantial workload of clinical teachers.

Mobile learning applications have further instigated a revolutionary transformation in nurse training (15). These apps present features like nursing knowledge databases, animated demonstrations of procedures, and daily quizzes. They facilitate new nurses in leveraging fragmented time for learning and offer a platform for the exchange of experiences and knowledge. Nevertheless, the efficacy of these apps hinges on the quality and evidence-based nature of the content. Research is requisite to validate the influence of mobile learning applications on the competency development of new nurses.

### 2.5 Teamwork training initiatives

Effective teamwork is of fundamental significance in healthcare settings. Interdisciplinary team training initiatives for new nurses entail collaboration with physicians, pharmacists, and other healthcare professionals. Via activities like case discussions and joint patient care planning, new nurses can acquire a more profound comprehension of the roles and obligations of other team constituents and refine their teamwork capabilities. A study conducted by Yeh et al. (16) revealed that such training programs augmented the communication and coordination proficiencies of new nurses, thereby resulting in enhanced patient outcomes. Teamwork simulation exercises, such as responding to a simulated ward fire, can also assist new nurses in cultivating practical skills within a team framework. Moreover, instituting a reward mechanism for teamwork can motivate new nurses to actively partake in collaborative undertakings.

### 2.6 Reflective practice and its significance

Reflective practice serves as a fundamental cornerstone for the professional development and growth of new nurses. Subsequent to clinical experiences, engaging in group discussions and maintaining reflective diaries can assist new nurses in dissecting their actions, pinpointing areas that warrant improvement, and deriving valuable learning from their experiences. For instance, in a qualitative research study conducted by Colman et al. (17), new nurses attested that reflective practice augmented their self-awareness and clinical judgment. The application of models such as the Perinatal Bereavement Care Training Program (PBCTP), which incorporates elements of reflective learning, has been demonstrated to effectively address the emotional and professional requisites of nurses within specific clinical domains (18).

### 2.7 Scenario simulation case competitions

Scenario simulation case competitions present a distinctive opportunity for new nurses to apply their acquired knowledge and skills within a competitive yet collaborative milieu. These competitions, which are founded on actual nursing cases, necessitate teams of new nurses to formulate and execute comprehensive nursing care plans. Research has demonstrated that such competitions have the capacity to enhance the clinical reasoning, communication, and teamwork capabilities of new nurses (19, 20). They also play a role in augmenting the motivation and fostering a sense of professional identity among new nurses. A case simulation was conducted based on the application for newly employed nurses, establishing a fundamental and universal language for delirium assessment and management and facilitating communication among medical staff. The research posits that scenario simulation represents an efficacious strategy for clinical skills training in infectious diseases (21). The cooperation within the actual ICU environment in Italy is conducive to effective experiential learning and heightens the familiarity of newly employed nurses with the environment and equipment, which is highly advantageous for the enhancement of their technical and non-technical skills (22).

### 2.8 Mentorship and social support

In numerous healthcare systems, mentorship programs assume a crucial role in the professional advancement of new nurses. Take South China as an instance, where social support and mentorship have been demonstrated to boost the self-efficacy and job satisfaction of new nurses (23). The Nurse Residency Training Program (NRTP), overseen by seasoned nurse educators, is capable of furnishing new nurses with structured backing and direction during their shift to clinical practice. Incorporating mentorship into residency programs can assist new nurses in acclimating to the organizational culture and cultivating the requisite competencies in a more efficient manner.

### 2.9 Comprehensive training approaches

Drawing upon an exhaustive literature review, semi-structured interviews, and questionnaire surveys centered around job competence, an online nurse training curriculum was devised and put into effect. This initiative has demonstrably augmented the comprehensive practical proficiencies and home care aptitudes of online nurses (24). The index framework for the standardized training of newly enlisted neurosurgery nurses, which was formulated through semi-structured interviews and the Delphi expert consultation technique, can function as a valuable benchmark for the specialized training of neurosurgery nurses during the standardized training phase (25). The research anchored in the ADDIE model, distinguished by its flexible modalities and salutary experiences, encompasses five sequential stages: analysis, design, development, implementation, and evaluation. In accordance with the actual clinical exigencies, the teaching content, teaching modality, and instructional hours were meticulously developed and executed, and the self-efficacy and core competencies of nurses were appraised (26). Through the integration of the workshop teaching paradigm and the virtual simulation practice methodology, a training regimen for newly recruited critical care nurses was contrived, thereby endowing the training program with enhanced allure and scientific rigor and laying a theoretical bedrock for the training of specialized nurses. The six-step standard communication protocol, which was conceived by Chinese scholars via the Chinese localization and refinement of the CICARE communication model, namely “connect - introduce - communicate - question - respond - exit” (27), was implemented. Based on this “six-step standard communication process,” a communication skills training program for newly employed nurses was constructed. This program assimilates the nursing idiosyncrasies and clinical requisites of cancer patients, streamlines and standardizes each interaction between nurses and patients. It serves as an efficacious communication stratagem to cultivate trust and engender harmony between nurses and patients. As a result, the professional caliber and communication skills of nurses have been expeditiously enhanced, and their faculty to dissect and resolve practical problems has been honed.

## 3 Key considerations in training implementation

### 3.1 Training needs assessment

Prior to the execution of any training program, a comprehensive and meticulous assessment of the training requisites of new nurses is of utmost significance. This process entails the collection of information regarding their educational backgrounds, extant knowledge and skillsets, as well as their career aspirations. Instruments such as questionnaires, interviews, and skills evaluations can be employed to amass this data. Grounded on the outcomes of the assessment, individualized training blueprints can be formulated to guarantee that the training is pertinent and efficacious. For instance, a study by Qia and Liu (28) underscored the criticality of needs assessment in customizing training programs to precisely meet the specific exigencies of new nurses.

### 3.2 Training staff development

The caliber of the training personnel directly exerts an influence on the efficacy of the training. Regardless of whether it pertains to traditional or contemporary training modalities, trainers are required to possess current and updated knowledge, along with outstanding pedagogical skills. Regular training sessions and professional development prospects ought to be furnished to the training staff to ensure their alignment with the most recent nursing practices and educational methodologies (29). Incentive frameworks, such as the acknowledgment and recompense for exemplary teaching, can also augment the motivation and job performance of the training staff.

### 3.3 Training effect evaluation

The establishment of a sound and comprehensive evaluation system is of cardinal importance for gauging the success of the training program. Evaluation metrics should encompass theoretical knowledge attainment, practical skills competence, patient satisfaction levels, and teamwork competencies. A multiplicity of evaluation techniques, including written examinations, clinical skills appraisals, and patient feedback questionnaires, can be employed to acquire a holistic comprehension of the training efficacy. In light of the evaluation outcomes, prompt modifications to the training approaches and content can be effected to perpetually refine and optimize the training program. For example, a research endeavor by Zhang et al. (30) and Shang et al. (31) formulated an evaluation index system for new nurse training and illustrated its application in enhancing the quality of the training regimen.

## 4 Challenges faced

Both the procurement of simulation training apparatus, the development and upkeep of information platforms, and the establishment of a high-caliber teaching faculty demand substantial resource allocation. In the case of certain small hospitals or primary medical institutions, financial constraints may emerge, thereby impeding the utilization of advanced training modalities.

Newly recruited nurses bear a substantial workload in clinical practice, and the judicious scheduling of training time poses a formidable challenge. An overly protracted training period might disrupt the normal work and rest patterns of new nurses and precipitate work-related exhaustion; conversely, an excessively brief training interval would compromise the attainment of training objectives. It is imperative to orchestrate the training schedule in a scientific manner, while ensuring the seamless progression of clinical operations.

The cultural milieu and organizational governance structure of a hospital can exert an influence on the implementation of training methodologies. Some hospitals may lack an organizational culture that fosters innovation and teamwork, potentially dampening the enthusiasm of new nurses toward novel training approaches. Concurrently, suboptimal organizational management could result in the ineffective execution of training plans and a significant diminution in training efficacy (32) reported that the introduction of the Japanese standardized clinical training system in the Vietnam pilot project augmented the capabilities of nurses and instigated organizational transformation, thereby exerting a salutary influence on nursing practices and the professional standing of nurses.

## 5 Conclusion

In order to enhance the job competency of newly hired nurses, it is essential to adopt a comprehensive approach by applying a diverse range of training methods. Continuous refinement and innovation of traditional training paradigms are required, along with the full exploitation of modern training techniques such as simulation training, information-driven training, and team collaboration training. During the training implementation process, particular attention should be given to training needs assessment, the integration of multiple methods, the development of the teaching workforce, and the evaluation of training outcomes. Challenges in areas such as resource allocation, time management, and cultural and organizational aspects must be surmounted to foster nursing professionals with high job competency, thereby providing a robust safeguard for elevating nursing quality and ensuring patient safety. Looking ahead, further exploration of more efficacious training methods and models remains necessary to keep pace with the perpetually evolving medical landscape and nursing requirements.
